# Caring for People With Dementia Under COVID-19 Restrictions: A Pilot Study on Family Caregivers

**DOI:** 10.3389/fnagi.2021.652833

**Published:** 2021-04-20

**Authors:** Elena Carbone, Rocco Palumbo, Alberto Di Domenico, Silvia Vettor, Giorgio Pavan, Erika Borella

**Affiliations:** ^1^Department of General Psychology, University of Padova, Padova, Italy; ^2^Department of Psychological, Health and Territorial Sciences, G. d’Annunzio University of Chieti-Pescara, Chieti, Italy; ^3^Istituto per Servizi di Ricovero e Assistenza agli Anziani, Treviso, Italy

**Keywords:** dementia, family caregivers, caregivers’ distress, behavioral and psychological symptoms of dementia (BPSD), loneliness, resilience, COVID-19 lockdown

## Abstract

**Introduction:**

The present pilot study examined to what extent the COVID-19 lockdown affected the behavioral and psychological symptoms of dementia (BPSD) in people with dementia and worsened their family caregivers’ distress. The associations between changes in the BPSD of relatives with dementia (RwD) and in their caregivers’ distress, and sense of social and emotional loneliness, and resilience were also investigated.

**Materials and Methods:**

Thirty-five caregivers of RwD attending formal healthcare services before the COVID-19 lockdown volunteered for the study, and were interviewed by phone during the lockdown. Caregivers completed the NeuroPsychiatric Inventory (NPI) to assess their care recipients’ BPSD and their own distress, and two questionnaires assessing their social and emotional loneliness, and their resilience.

**Results:**

No clear changes emerged in either the BPSD of the RwD or the caregivers’ distress during lockdown compared with before the pandemic. Caregivers reporting more frequent and severe BPSD in their RwD before the lockdown scored higher on emotional loneliness. Those reporting more frequent and severe BPSD under lockdown, especially men and those taking care of RwD with more advanced dementia, scored higher on both social and emotional loneliness. A significant negative correlation also emerged between caregivers’ resilience and changes in their level of distress due to the lockdown, with female caregivers reporting greater resilience.

**Discussion:**

Our findings offer preliminary insight on the effects of loneliness and resilience, and on the influence of individual characteristics on the experience and consequences of informal caregiving for RwD in times of restrictions imposed by a pandemic.

## Introduction

People with dementia (PwD) living at home depend largely on family (informal) caregivers for assistance. Caring for a person with dementia can cause severe psychological morbidity, stress, and emotional burden in family caregivers (Pinquart and Sörensen, 2003). The availability of a network of social and healthcare services (day centers, home-based interventions of healthcare professionals, etc.) plays an important part in helping caregivers to dealing with the day-to-day care of a relative with dementia (RwD), and in managing their stressful role (Jensen et al., 2015; Brooks et al., 2018; Lobbia et al., 2019; Carbone et al., in press).

As a result of the “stay-at-home” rules prompted by the COVID-19 pandemic, family members caring for RwD have had to cope with disrupted daily routines, a lesser availability of formal healthcare services for themselves and their RwD, impoverished social relations, and less contact with loved ones. Studies assessing the impact of the COVID-19 lockdown on the behavioral and psychological symptoms of dementia (BPSD) in community-dwelling people with PwD, and on their family caregivers’ distress have suggested a worsening of BPSD in PwD under lockdown restrictions with their agitation, apathy, and depression emerging as the most affected symptoms (Cagnin et al., 2020; Canevelli et al., 2020; Lara et al., 2020). Caregivers also reported feeling a greater emotional burden (Cohen et al., 2020) and psychological stress (Cagnin et al., 2020), with worsening levels of anxiety, helplessness, irritability and depression (Cagnin et al., 2020).

However, most of these studies had no baseline assessment obtained before the pandemic for comparison. Asking caregivers to rate whether they experienced changes in the BPSD of their relatives, and in their own related burden and distress during lockdown might have led them to overestimate their difficulties and stress-related feelings. The one study (Lara et al., 2020) that compared a baseline assessment obtained before the pandemic with one conducted under lockdown focused only on caregivers’ perceptions of changes in the BPSD of their care recipients, without considering any changes in their own levels of distress.

Little is known about the influence of potential exacerbating or protective factors—such as social and emotional loneliness or resilience (as outlined below)—on caregivers’ perceptions of changes in the BPSD of PwD, and their own related distress, in such an unexpected and prolonged stressful situation as a lockdown.

The aim of the present pilot study was therefore to explore changes due to the COVID-19 lockdown in the BPSD of community-dwelling PwD and the distress experienced by their family caregivers. To do so, a well-known and widely used scale for assessing BPSD as perceived by caregivers of individuals with dementia was used, i.e., the NeuroPsychiatric Inventory (NPI; Cummings et al., 1994). This tool is a questionnaire administered to caregivers, and can be completed by phone. In order to obtain a “baseline” assessment of caregivers’ perceptions of the frequency and severity of BPSD in their RwD, and of their own related distress, we considered dyads of informal caregivers and care recipients being monitored by territorial services. This enabled us to capture any differences in the BPSD of the RwD and/or in the distress perceived by the caregiver during lockdown compared with the situation beforehand.

Another aim was to newly explore the associations between caregivers’ ratings of the frequency and severity of their relative’s BPSD and of their own related distress (both in their usual situation and under lockdown), any changes in these ratings due to lockdown (in terms of worsening BPSD and caregiver distress levels) and caregivers’ perceived social and emotional loneliness, and resilience, i.e., the ability to cope with adversity, and adapt to the physical and psychological challenges of caregiving (Teahan et al., 2018). We concentrated on loneliness and resilience because of their relevance to caregiving. Feeling socially and emotionally lonely is known to affect caregivers’ health and wellbeing (Victor et al., in press), thereby influencing the quality of the care they can provide for their RwD. On the other hand, resilience has proved to protect against the fallout of caregiving on an individual’s health and psychological wellbeing (Teahan et al., 2018), under COVID-19 lockdown as well (Altieri and Santangelo, 2020).

In line with previous evidence (e.g., Park, 2020), we expected to find changes (a worsening) in BPSD due to the lockdown, as well as an exacerbation of caregivers’ distress.

As for social and emotional loneliness, its negative effect on caregivers’ health and wellbeing (Victor et al., in press) is well known, and may have been exacerbated by the restrictions imposed to combat the COVID-19 pandemic (Smith and Lim, 2020). We therefore expected caregivers who reported more severe social and emotional loneliness to perceive more frequent and severe BPSD in their RwD, and more distress in themselves as a consequence, as well as greater changes in these aspects (in the sense of a further deterioration) due to the lockdown. As concerns resilience, in line with previous evidence of its protective role for caregiving (Altieri and Santangelo, 2020), we expected a greater degree of resilience to be associated with lower caregiver ratings, and fewer reported lockdown-related changes in the frequency and severity of the BPSD in their RwD, and in their own related distress.

Other well-known factors influencing the frequency and severity of BPSD in PwD and the subsequent psychological consequences experienced by family caregivers include gender—of both the caregiver (Lin et al., 2012) and the person with dementia—and the stage of dementia (Connell et al., 2001). These factors were therefore also considered to ascertain their associations with caregivers’ NPI ratings in normal times and under lockdown (and any differences between the two), and also with caregivers’ perceived loneliness and resilience.

## Materials and Methods

### Participants

The study involved 35 family volunteer caregivers of community-dwelling PwD who had been monitored by an institute providing residential and non-residential care services for the elderly [*Istituto per Servizi di Ricovero e Assistenza agli Anziani* (ISRAA)] in Treviso, Italy for at least 18 months before the pandemic.

Before the pandemic, a routine assessment had been conducted for all dyads, which involved staging their condition with the Clinical Dementia Rating (CDR; Hughes et al., 1982), and scoring their neuropsychiatric symptoms with the NPI, which was completed by their caregivers.

Table 1 shows the demographic characteristics of the caregivers and their RwD. Almost all caregivers (*N* = 34) were family members (spouses, children, or siblings), while one was a paid living-in carer. The PwD had been diagnosed with Alzheimer’s disease in 17.1% of cases, with vascular dementia in 37.1%, and with mixed or other types of dementia in 60%. Disease severity according to the CDR was low-moderate in 62.8% of cases, and severe in 37.14%.

**TABLE 1:** Demographic characteristics of family caregivers and their relatives with dementia.

	Caregivers *N* = 35	People with dementia *N* = 35
Mean (SD) age, years	61.23 (9.91)	82.60 (8.91)
Women, *n* (%)	26 (74.28%)	22 (62.9%)

Family caregivers agreed to complete a telephone interview during a period of lockdown prompted by the COVID-19 pandemic (in May and early June, 2020).

### Materials

#### NeuroPsychiatric Inventory (Cummings et al., 1994).

NeuroPsychiatric Inventory tool is used to assess 12 behavioral and psychological symptoms in PwD. For each symptom, caregivers are asked to rate its frequency and severity and the emotional and psychological distress experienced. During the telephone interview, caregivers were asked to answer the questions in the NPI regarding their RwD and themselves, focusing on the previous month (i.e., under lockdown).

The dependent variables were: (i) the sum of the frequency X severity scores (total NPI score for care recipient BPSD), and the frequency X severity scores for each symptom and (ii) the sum of the caregivers’ distress scores (total NPI caregiver distress score), and the caregivers’ distress scores regarding each symptom. Higher scores indicated more frequent and severe BPSD, and more severe caregiver distress.

#### Social and Emotional Loneliness Scale (De Jong and Van Tilburg, 2006)

Social and Emotional Loneliness scale tool comprises six items for assessing emotional loneliness, and social loneliness. During the telephone interview, caregivers were asked to rate their agreement with each item from 1 (absolutely true) to 5 (absolutely not true) regarding the previous month (i.e., during lockdown).

The dependent variables were the sum of the scores for the three items for emotional loneliness and the three items for social loneliness, with lower scores indicating higher levels of social and emotional loneliness.

#### Connor-Davidson Resilience Scale—10 Items (Campbell-Sills and Stein, 2007)

Connor-Davidson Resilience scale comprises 10 items measuring the ability to cope with adversity. During the telephone interview, caregivers were asked to rate each item from 0 (not true at all) to 4 (true nearly all the time) in relation to the previous month (i.e., under lockdown). The dependent variable was the sum of the scores for the 10 items, with higher scores corresponding to a greater resilience.

### Procedure

Family caregivers were contacted by phone and completed a single interview lasting about 45 min. Interviews were conducted by the ISRAA psychologists between May and early June, 2020. After obtaining their consent, the experimenter guided participants through their completion of the questionnaires in the following order: NPI; Social and Emotional Loneliness scale; Resilience Scale.

### Statistical Analyses

First, any changes between the reported BPSD and caregivers’ distress before and during the lockdown were examined. Given the small sample size and the non-normal distributions (analyzed with the Shapiro–Wilk test) of the NPI ratings of the frequency and severity of dementia symptoms, and of the caregivers’ distress, the changes in NPI scores were analyzed using the Wilcoxon test for paired samples.

Then two indexes of the changes in NPI scores were computed to test whether caregivers’ social and emotional loneliness, and their resilience under lockdown respectively exacerbated or buffered their perceptions of any changes in the frequency and severity of BPSD in their RwD, and in their own related distress. These two indexes concerned the change in the total NPI (i) scores for care recipient BPSD (the total NPI score during lockdown minus the total NPI score before the pandemic) and (ii) caregiver distress scores (the total NPI caregiver distress score during lockdown minus the total NPI caregiver distress score before the pandemic).

Spearman’s correlations were run between these NPI change indexes, along with the NPI scores at the baseline (before the pandemic) and during lockdown, and the scores on the measures of Social and Emotional Loneliness, and Resilience. The relationships between gender (of both caregiver and the RwD) and dementia stage with the NPI scores at the baseline and under lockdown, the two indexes of the changes in NPI scores, and the caregivers’ ratings of their own social and emotional loneliness and resilience were also ascertained.

## Results

Descriptive statistics for the NPI scores by time of assessment (before the pandemic versus during lockdown) are shown in Table 2.

**TABLE 2:** Descriptive statistics for the NPI scores for behavioral and psychological symptoms (frequency X severity) in people with dementia, and for their caregivers’ distress, by time of assessment (before the pandemic and during lockdown).

	NPI scores for care recipient BPSD (frequency X severity)				NPI caregiver distress scores			
	Before pandemic		During lockdown		Before pandemic		During lockdown	
	*M*	SD	*M*	SD	*M*	SD	*M*	SD
Total score	31.51	14.23	30.68	15.29	20.08	8.82	19.65	7.94
Delirium	3.69	3.57	2.91	3.16	2.40	2.00	2.09	1.83
Hallucinations	1.37	2.54	1.77	2.90	0.91	1.56	1.14	1.75
Agitation	4.94	3.23	4.80	3.28	3.03	1.67	2.89	1.84
Depression	1.74	2.63	1.91	2.80	1.17	1.59	1.37	1.80
Anxiety	4.00	3.69	3.57	3.64	2.40	2.00	2.26	1.93
Dysphoria	0.20	0.58	0.20	0.67	0.20	0.58	0.14	0.60
Apathy	2.51	2.90	2.74	3.22	1.71	1.85	1.77	1.89
Disinhibition	1.14	2.42	1.03	2.38	0.74	1.42	0.77	1.55
Irritability	2.34	2.62	2.26	2.91	1.69	1.79	1.49	1.73
Motion	4.97	4.16	4.34	3.91	2.91	2.16	2.49	2.10
Sleep	3.46	3.48	3.37	3.80	2.14	2.08	2.00	2.12
Food	1.14	2.30	1.77	2.47	0.77	1.53	1.26	1.63

*NPI, NeuroPsychiatric Inventory; BPSD, behavioral and psychological symptoms of dementia.*

Concerning any changes in the BPSD of PwD, no significant differences emerged, neither between their total NPI scores at the baseline and during lockdown (*Z* = −0.50; *p* = 0.61), nor when each of the symptoms included in the NPI were considered.

As for changes in caregivers’ distress, there were no significant differences, neither between their total NPI distress scores at the baseline and during lockdown (*Z* = −0.61; *p* = 0.54), nor in relation to each of the symptoms included in the NPI (see Table 2).

To further investigate any lockdown-induced changes in BPSD and caregivers’ distress, the NPI scores were analyzed qualitatively at individual level. We analyzed whether any caregivers’ scores that changed between the two assessments pointed to a deterioration (higher scores) or an improvement (lower scores) in their NPI ratings under lockdown by as much as 1 SD with respect to the mean score at the baseline for the sample as a whole. The total NPI scores (see Table 3) attributed by the caregivers under lockdown indicated that the frequency and severity of the BPSD in their RwD remained the same in 74.28% of cases, worsened in 14.28%, and improved in 11.42%. The total NPI caregiver distress scores indicated that, under lockdown, 85.7% of caregivers experienced no change in their levels of distress, 11.42% became more distressed and 2.85% became less so.

**TABLE 3:** Proportions of caregivers with NPI scores rising (i.e., worsening) or falling (i.e., improving) by 1 SD, or remaining the same under lockdown compared with before the pandemic.

	Rising (%)	Unchanged (%)	Falling (%)
Total NPI scores for care recipients’ BPSD	14.28	74.28	11.42
Total NPI caregivers distress score	11.42	85.71	2.85

*NPI, NeuroPsychiatric Inventory; BPSD, behavioral and psychological symptoms of dementia.*

Significant and moderate correlations emerged between the scores obtained with the NPI, and on the Social and Emotional Loneliness, and Resilience scales (see Table 4): (i) between social loneliness and the total NPI score for the RwD during the lockdown; (ii) between emotional loneliness and the total NPI scores for the RwD, both before and during the lockdown; and (iii) between resilience and changes in total NPI caregiver distress scores, indicating that a greater resilience was associated with a more limited worsening under lockdown of the distress experienced by caregivers regarding the BPSD of their RwD.

**TABLE 4:** Correlations between the NeuroPsychiatric Inventory scores, the Social and Emotional Loneliness scale, and the Resilience scale.

	1	2	3	4	5	6	7	8
1. Change in total NPI score for care recipient BPSD	–							
2. Change in total NPI caregiver distress score	0.66**	–						
3. Total NPI score for care recipient BPSD (before pandemic)	–0.24	−0.45**	–					
4. Total NPI score for care recipient BPSD (during lockdown)	0.31*	–0.02	0.80**	–				
5. Total NPI caregiver distress score (before pandemic)	–0.15	−0.54**	0.91**	0.74**	–			
6. Total NPI caregiver distress score (during lockdown)	0.31*	0.10	0.71**	0.89**	0.73**	–		
7. Emotional loneliness	–0.15	0.11	−0.30*	−0.37*	–0.26	–0.27	–	
8. Social loneliness	–0.12	0.08	–0.27	−0.36*	–0.26	–0.22	0.51**	–
9. Resilience	–0.20	−0.32*	0.05	–0.08	0.04	–0.13	–0.11	0.01

*N* = 35. NPI, NeuroPsychiatric Inventory; BPSD, behavioral and psychological symptoms of dementia. *p < 0.05. **p < 0.01.

As for the associations between caregiver and RwD gender, stage of dementia and the measures of interest, significant correlations emerged between caregivers’ gender and both NPI total scores (*r* = −0.33, *p* < 0.05) and NPI distress total scores (*r* = −0.39, *p* < 0.01) under lockdown, and the Resilience scale (*r* = 0.32, *p* < 0.05). Female caregivers reported lower ratings for the frequency and severity of BPSD in their RwD and for their own distress under lockdown, and higher ratings for their own resilience.

Significant correlations emerged between stage of dementia (CDR) and NPI total scores under lockdown only (*r* = 0.31, *p* < 0.05), a more severe stage of dementia being associated with a higher frequency and severity of BPSD under lockdown. A more severe stage of dementia was also associated with higher caregivers’ ratings of their own emotional (*r* = −0.48, *p* < 0.01) and social (*r* = −0.29, *p* < 0.05) loneliness.

A significant correlation was then found between the gender of the PwD and the caregivers’ perceived emotional loneliness (*r* = 0.33, *p* < 0.05), with caregivers of female RwD reporting lower rates of emotional loneliness.

## Discussion

The aim of the present study was to further explore the impact of the COVID-19 lockdown on BPSD in community-dwelling PwD, and the related distress experienced by their informal caregivers. Compared with other COVID-19 literature, a strength of this study lies in the availability of a baseline assessment (before the pandemic) of caregivers’ perceptions of the BPSD of the person in their care, and of their own related distress, that could be compared with the assessment conducted under lockdown. Any associations between changes in care recipients’ BPSD and the related emotional and psychological consequences (distress) experienced by their caregivers on the one hand, and the latter’s social and emotional loneliness, and resilience on the other were also newly ascertained.

Contrary to our expectations, lockdown was generally not associated with any significant deterioration neither in the frequency and severity of any of the BPSD in the PwD nor in their caregivers’ levels of distress. There may be some reasons why this pattern of findings seems to contrast with previous evidence (e.g., Park, 2020). For a start, as caregiving is a stressful situation that imposes physical, mental and social constraints, it may be that the lockdown to stem the COVID-19 pandemic had little or no additional impact on our caregivers’ routines. However, our caregivers had been supported, up until the start of the pandemic at least, by a formal network of healthcare services, i.e., attended psychoeducational or support group meetings, and had their RwD participating in cognitive stimulation programs, which are known to have a key role in providing caregivers with strategies and resources to cope with day-to-day care for a person with dementia (Jensen et al., 2015; Brooks et al., 2018; Lobbia et al., 2019; Carbone et al., in press). These aspects could have attenuated any negative consequences of lockdown on the latter’s dementia symptoms and on their own distress. These are only speculations, but they do seem to be corroborated by the general lack of any lockdown-induced changes at individual level in caregivers’ ratings of their care recipients’ BPSD and of their own distress. Only a minority (14.28%) of our caregivers reported more frequent and severe BPSD in their RwD, and in their own related distress (in 11.42% of cases) under lockdown than at the baseline. Few of them also reported a decrease in the care recipients’ BPSD or their own distress under lockdown. A more severe stage of dementia was found associated with worse BPSD under lockdown, in line with previous studies (e.g., Cagnin et al., 2020), however.

The availability of a baseline assessment obtained before the pandemic may have helped us to clarify whether lockdown influence the BPSD of PwD and their caregivers’ distress. Previous studies (e.g., Cagnin et al., 2020; Canevelli et al., 2020) that only considered caregivers’ ratings obtained during lockdown may therefore have led to an overestimation of their difficulties and stress-related feelings.

A more nuanced picture of how our caregivers might have experienced such a prolonged stressful situation as the lockdown seems to come from examining their perceived social and emotional loneliness, and their resilience under lockdown restrictions. Somewhat contrary to our expectations, no significant associations emerged between caregivers’ perceived emotional or social loneliness and any changes in the BPSD of their care recipient or their own related distress. Our results thus indicate that, while caregiver reporting more severe emotional loneliness also reported more frequent and severe BPSD in their RwD both before and during lockdown, any reportedly more severe social loneliness was only associated with worse symptoms in the person with dementia during lockdown. Such a pattern of findings suggests that feeling less emotionally supported (i.e., a lack of deep and meaningful relationships) broadly affects one of the primary sources of caregiver stress (Gaugler et al., 2000), which is the way in which they experience and cope with their care recipients’ BPSD. This is true in normal times, and also in such stressful situations as a lockdown. Perceiving the absence of a social support network seemed instead to particularly affect caregivers’ perceptions of their care recipients’ BPSD during lockdown. Previous studies also suggested that being able to rely on an informal support network during lockdown (e.g., living with other family members or getting help from other relatives) helped caregivers to deal with their care recipients’ needs, so the former experienced lower overload (Savla et al., 2020) and were less stressed (Cagnin et al., 2020) by their role as carers. It might be argued that loneliness, which is also conceived as a fairly stable individual characteristic (Mund et al., 2020), has a broadly adverse effect on caregiving (Teahan et al., 2018). A challenging situation like lockdown, which causes a further impoverishment of formal and informal social contacts, exacerbates the negative effects of caregivers’ perceptions of a lack of social support (i.e., their sense of social loneliness) on their ratings of the dementia symptoms of the person in their care. Interestingly, a more severe stage of dementia was found associated with caregivers’ higher ratings of social and emotional loneliness, suggesting that those taking care of people with more severe symptoms felt more emotionally and socially lonely in such an unexpected and overwhelming situation as COVID-19 lockdown. The impact of the stage of dementia on the demands of caregiving is well known, but we could not say whether it was worsened by living under lockdown. Having assessed our caregivers’ social and emotional loneliness before the pandemic would have helped us to clarify this issue, which seems worth investigating.

Again going against our expectations, we found no associations between caregivers’ resilience and the NPI ratings at the baseline or under lockdown, neither with the caregivers’ ratings of the BPSD in their RwD and their own distress, nor with any changes in the frequency and severity of BPSD between the two assessments. Here again, this might be due to the above-mentioned fact that our caregivers could normally (before the pandemic) rely on a network of healthcare services that helped them to acquire strategies and resources for managing the challenges of caring for a RwD. This may have made them more adaptable and better able to manage their day-to-day caring for a person with dementia even when such an unexpected situation as the COVID-19 lockdown meant that such formal healthcare services for themselves and their RwD were much less available. Nevertheless, our index of the changes in the NPI scores for caregiver distress showed that greater resilience was associated with smaller changes (i.e., a more limited worsening) in caregivers’ distress during lockdown. This result suggests that, faced with an unexpected and challenging event like lockdown, caregivers who perceived themselves as capable of engaging recourses to adapt to such stressful situations did not experience any increase in the emotional and psychological distress elicited by the BPSD of their RwD. However, our female caregivers returned higher ratings for their resilience, lower ratings for both the frequency and the severity of BPSD in their RwD, and lower ratings for the distress they themselves experienced under lockdown. Although female gender seems to be generally associated with negative experiences and a higher burden of care for RwD (Pinquart and Sörensen, 2006), a possible explanation for this pattern of results may lie in that the changes in these female caregivers’ daily routines imposed by the lockdown left them with more time and resources to spend on their relative’s needs under lockdown. They would thus experienced less distress and feel better able to adapt and cope with the challenges of caregiving even in such extraordinary circumstances. In most cases, caregiving is stressful *per se*, and the stress it causes might mitigate the influence of another stressful situation like the COVID-19 emergency. These results broadly underscore how resilience can buffer the negative consequences of caring for a person with dementia on caregivers’ psychological and emotional functioning, even under stressful conditions like those prompted by a pandemic.

Despite these interesting findings, our study has some limitations. First of all, the study involved only a small sample of caregivers. Although power analysis (G^∗^Power) showed that a sample of 35 participants sufficed to attain a power of.80 in a repeated-measures within-participant comparison with a medium effect size and a significance level of α = 0.05, our results should be interpreted with caution. Unlike the self-administered surveys, using the NPI made it necessary to interview family caregivers directly, which is time-consuming for the caregivers themselves (e.g., Lara et al., 2020).

The present work could therefore be seen as a pilot study of potential use in informing further research on the issues discussed here, since the ongoing COVID pandemic might still be an issue for families caring for RwD.

Second, we interviewed our participants at the very end of the first lockdown, but repeated monitoring at different stages of the lockdown, or of the ongoing pandemic, might have given us a better picture of any changes in caregivers’ perceptions of the frequency and severity of BPSD in their care recipient, and their own related distress.

Besides the effects of gender and stage of dementia, other variables of potential interest relating, for instance, to the person with dementia (level of dependence), the caregiver (personality traits), and the caregiving role (amount of formal and informal support received) were not considered here. Jointly considering all these aspects might help to clarify the impact of lockdown conditions on the BPSD of individuals living with dementia, their emotional and psychological fallout on their caregivers’ distress, and also the influence of caregivers’ perceived social and emotional loneliness and resilience.

Overall, although we found no clear impact of lockdown on the BPSD of PwD or the related distress perceived by their caregivers, our findings offer helpful insight on how an extraordinary situation like lockdown, that imposed physical and social distancing, and interfered with the support usually provided by formal healthcare services affected the informal caregiving of PwD. In such conditions, caring for a RwD might become even more stressful than usual, especially for caregivers who feel emotionally and socially lonely, and lacking in the resources needed to cope with the challenges of caregiving. A more complex picture might also emerge when individual characteristics of the PwD and/or their family caregivers are taken into account. Even in the time of a pandemic, it seems important to ensure the continuity of healthcare services for dementia, and the availability of a social support network for informal caregivers (including virtual psychoeducational programs, for instance, that could be adjusted to the specific needs and characteristics of a given caregiver and the person in their care).

## Data Availability Statement

The raw data supporting the conclusions of this article will be made available by the authors, without undue reservation.

## Ethics Statement

The studies involving human participants were reviewed and approved by Ethical Committee for the Psychological Research, University of Padova, Padova, Italy. The patients/participants provided their written informed consent to participate in this study.

## Author Contributions

EC contributed in analyzing and interpreting the data and in writing the manuscript. RP and AD contributed in writing the manuscript. SV and GP contributed in enrolling participants and supervised the data collection. EB designed the study and contributed in analyzing and interpreting the data and in writing the manuscript. All authors read and approved the final manuscript.

## Conflict of Interest

The authors declare that the research was conducted in the absence of any commercial or financial relationships that could be construed as a potential conflict of interest.

## References

[B1] Altieri M. Santangelo G. (2020). The psychological impact of COVID-19 pandemic and lockdown on caregivers of people with dementia. *Am. J. Getriatr. Psychiatr.* 29 27–34. 10.1016/j.jagp.2020.10.009 33153872 PMC7577876

[B2] Brooks D. Fielding E. Beattie E. Edwards H. Hines S. (2018). Effectiveness of psychosocial interventions on the psychological health and emotional well-being of family carers of people with dementia following residential care placement: a systematic review. *JBI Evid. Synth.* 16 1240–1268. 10.11124/JBISRIR-2017-003634 29762315

[B3] Cagnin A. Di Lorenzo R. Marra C. Bonanni L. Cupidi C. Laganà V. (2020). Behavioral and psychological effects of coronavirus disease-19 quarantine in patients with dementia. *Front. Psychiatry* 11:578015. 10.3389/fpsyt.2020.578015 33033486 PMC7509598

[B4] Campbell-Sills L. Stein M. B. (2007). Psychometric analysis and refinement of the Connor–Davidson Resilience Scale (CD-RISC): validation of a 10-item measure of resilience. *J. Trauma Stress* 20 1019–1028. 10.1002/jts.20271 18157881

[B5] Canevelli M. Valletta M. Blasi M. T. Remoli G. Sarti G. Nuti F. (2020). Facing dementia during the COVID-19 outbreak. *J. Am. Geriatr. Soc.* 68 1673–1676. 10.1111/jgs.16644 32516441 PMC7300919

[B6] Carbone E. Gardini S. Pastore M. Piras F. Vincenzi M. Borella E. (in press). Cognitive Stimulation Therapy (CST) for older adults with mild-to-moderate dementia in Italy: effects on cognitive functioning, and on emotional and neuropsychiatric symptoms. *J. Gerontol. B Psychol. Sci. Soc. Sci.* 10.1093/geronb/gbab007 33437991

[B7] Cohen G. Russo M. J. Campos J. A. Allegri R. F. (2020). Living with dementia: increased level of caregiver stress in times of COVID-19. *Int. Psychogeriatr.* 30 1–5. 10.1017/S1041610220001593 32729446 PMC7453351

[B8] Connell C. M. Janevic M. R. Gallant M. P. (2001). The costs of caring: impact of dementia on family caregivers. *J. Geriatr. Psychiatry Neurol.* 14 179–187. 10.1177/089198870101400403 11794446

[B9] Cummings J. L. Mega M. Gray K. Rosenberg-Thompson S. Carusi D. A. Gornbein J. (1994). The Neuropsychiatric Inventory: comprehensive assessment of psychopathology in dementia. *Neurology* 44 2308–2308. 10.1212/WNL.44.12.2308 7991117

[B10] De Jong J. Van Tilburg T. (2006). A 6-item scale for overall emotional, and social loneliness. Confirmatory tests on survey data. *Res. Aging* 28 582–598. 10.1177/0164027506289723

[B11] Gaugler J. E. Davey A. Pearlin L. I. Zarit S. H. (2000). Modeling caregiver adaptation over time: the longitudinal impact of behavior problems. *Psychol. Aging* 15 437–450. 10.1037/0882-7974.15.3.437 11014707

[B12] Hughes C. P. Berg L. Danziger W. Coben L. A. Martin R. L. (1982). A new clinical scale for the staging of dementia. *Br. J. Psychiatry* 140 566–572. 10.1192/bjp.140.6.566 7104545

[B13] Jensen M. Agbata I. N. Canavan M. McCarthy G. (2015). Effectiveness of educational interventions for informal caregivers of individuals with dementia residing in the community: systematic review and meta-analysis of randomised controlled trials. *Int. J. Geriatr. Psychiat.* 30 130–143. 10.1002/gps.4208 25354132

[B14] Lara B. B. Carnes A. Dakterzada F. Benitez I. Piñol-Ripoll G. (2020). Neuropsychiatric symptoms and quality of life in Spanish Alzheimer’s disease patients during COVID-19 lockdown. *Eur. J. Neurol.* 27 1744–1747. 10.1111/ene.14339 32449791 PMC7283827

[B15] Lin I. F. Fee H. R. Wu H. S. (2012). Negative and positive caregiving experiences: a closer look at the intersection of gender and relationship. *Fam. Relat.* 61 343–358. 10.1111/j.1741-3729.2011.00692.x 22544989 PMC3335398

[B16] Lobbia A. Carbone E. Faggian S. Gardini S. Piras F. Spector A. (2019). The efficacy of cognitive stimulation therapy (CST) for people with mild-to-moderate dementia. *Eur. Psychol.* 24 257–277. 10.1027/1016-9040/a000342

[B17] Mund M. Freuding M. M. Möbius K. Horn N. Neyer F. J. (2020). The stability and change of loneliness across the life span: a meta-analysis of longitudinal studies. *Pers. Soc. Psychol. Rev.* 24 24–52. 10.1177/1088868319850738 31179872 PMC6943963

[B18] Park S. S. (2020). Caregivers’ mental health and somatic symptoms during COVID-19. *J. Gerontol.* 76 e235–e240. 10.1093/geronb/gbaa121 32738144 PMC7454918

[B19] Pinquart M. Sörensen S. (2003). Differences between caregivers and non-caregivers in psychological health and physical health: a meta-analysis. *Psychol. Aging* 18 250–267. 12825775 10.1037/0882-7974.18.2.250

[B20] Pinquart M. Sörensen S. (2006). Gender differences in caregiver stressors, social resources, and health: an updated meta-analysis. *J. Gerontol. B Psychol. Sci. Soc. Sci.* 61 33–45. 10.1093/geronb/61.1.p33 16399940

[B21] Savla J. Roberto K. A. Blieszner R. McCann B. R. Hoyt E. Knight A. L. (2020). Dementia caregiving during the “stay-at-home” phase of COVID-19 pandemic. *J. Gerontol.* 76 e241–e245. 10.1093/geronb/gbaa129 32827214 PMC7546083

[B22] Smith B. J. Lim M. H. (2020). How the COVID-19 pandemic is focusing attention on loneliness and social isolation. *Public Health Res. Pract.* 30:e3022008. 10.17061/phrp3022008 32601651

[B23] Teahan Á. Lafferty A. McAuliffe E. Phelan A. O’Sullivan L. O’Shea D. (2018). Resilience in family caregiving for people with dementia: a systematic review. *Int. J. Getriatr. Psychiatr.* 33 1582–1595. 10.1002/gps.4972 30230018

[B24] Victor C. R. Rippon I. Quinn C. Nelis S. M. Martyr A. Hart N. (in press). The prevalence and predictors of loneliness in caregivers of people with dementia: findings from the IDEAL programme. *Aging Ment. Health.* 10.1080/13607863.2020.1753014 32306759

